# Effectiveness of Occlusal Splint Therapy in Moderating Temporomandibular Joint Disorders With Joint Displacement: A Retrospective Analysis Using Cone Beam Computed Tomography

**DOI:** 10.7759/cureus.57300

**Published:** 2024-03-31

**Authors:** Abin Mathew, Vivek CR, Alexander KA, Dwijesh Goswami, Tony Antony, Rakhi Bharat

**Affiliations:** 1 Department of Orthodontics and Dentofacial Orthopaedics, Grace Dental Care, Kottayam, IND; 2 Department of Orthodontics and Dentofacial Orthopaedics, Maaruti College of Dental Sciences & Research Centre, Bengaluru, IND; 3 Department of Orthodontics and Dentofacial Orthopaedics, Modern Smile Care Clinic & Medical Centre, Trivandrum, IND; 4 Department of Orthodontics and Dentofacial Orthopaedics, Ahmedabad Dental College & Hospital, Ahmedabad, IND; 5 Department of Orthodontics and Dentofacial Orthopaedics, Ambookens Specialty Dental Clinic, Kochi, IND; 6 Department of Orthodontics and Dentofacial Orthopaedics, Index Institute of Dental Sciences, Indore, IND

**Keywords:** orthodontics, condylar position, cone beam computed tomography, occlusal splint therapy, temporomandibular joint disorders

## Abstract

Background

Temporomandibular joint disorders (TMD) represent a prevalent group of conditions impacting the temporomandibular joint. Among the therapeutic interventions, occlusal splint therapy has gained recognition for its potential to address TMD symptoms, particularly in cases involving joint displacement.

Objective

This study aims to investigate the effectiveness of occlusal splint therapy in cases of moderate TMD with joint displacement, focusing on changes in condylar position, joint morphology, and patient-reported outcomes.

Methods

A retrospective analysis was conducted involving 148 participants who underwent occlusal splint therapy between January 2018 and December 2020. Data were collected through cone beam computed tomography (CBCT) imaging for precise assessments of condylar position and joint morphology. Ethical approval was obtained, and participants provided informed consent. Baseline characteristics, medical history, and TMD severity were recorded. Occlusal splint therapy included individualized fabrication, occlusal analysis, adjustments for optimal fit, and prescribed wear schedules. Follow-up included CBCT scans at specified intervals (three months and six months), with participant-reported outcomes collected. The data analysis was conducted using IBM SPSS Statistics for Windows, Version 22.0 (Released 2013; IBM Corp., Armonk, NY, USA). Paired t-tests or nonparametric equivalents were employed to assess changes in condylar position and joint morphology. Subgroup analyses were conducted to explore potential factors influencing treatment outcomes. The significance level was set at p < 0.05 for all statistical tests.

Results

The entire cohort (n = 148) had a mean age of 32.5 years (± 8.1), with a balanced gender distribution. Changes in condylar position revealed a statistically significant improvement (p = 0.03), with a mean decrease of 0.2 mm posttreatment. Joint morphology changes indicated increased joint space width (p = 0.01), improved disc position (p = 0.02), and nonsignificant alterations in bony structures (p = 0.10). Patient-reported outcomes demonstrated significant improvements in pain levels, jaw functionality, and satisfaction (all p < 0.001). Age and gender subgroup analyses showed consistent improvements in condylar position, joint morphology, and patient-reported outcomes across different groups.

Conclusion

Occlusal splint therapy demonstrated effectiveness in improving condylar position, joint morphology, and patient-reported outcomes in cases of moderate TMD with joint displacement. The findings underscore the potential of occlusal splint therapy as a viable intervention for managing TMD, providing valuable insights for clinicians and researchers.

## Introduction

Temporomandibular joint disorders (TMD) constitute a multifaceted and prevalent group of conditions affecting the temporomandibular joint (TMJ) and associated structures, affecting 25% of the global population [1,2]. These disorders encompass a spectrum of symptoms, including but not limited to pain, limited jaw movement, joint sounds, and compromised oral functions [3]. TMD can significantly impact an individual’s quality of life, leading to discomfort, disability, and psychosocial distress [4].

Among the diverse therapeutic modalities for TMD, occlusal splint therapy has garnered attention as a noninvasive intervention aimed at addressing specific manifestations of TMD, particularly those involving joint displacement [5]. Occlusal splints, also known as bite guards or night guards, are custom-fitted oral appliances designed to optimize occlusal relationships, distribute forces evenly, and reduce parafunctional habits [6]. The rationale behind occlusal splint therapy lies in its potential to influence condylar position, joint morphology, and overall TMJ function [7].

However, despite its widespread use and clinical efficacy, there remains a gap in understanding of the precise effects of occlusal splint therapy on condylar position, joint morphology, and patient-reported outcomes, especially in cases of moderate TMD with joint displacement. This study aims to provide a thorough understanding of the therapeutic benefits of occlusal splint therapy by employing reliable methodology, which includes larger sample sizes, standardized outcome measures, and longer follow-up periods. Addressing these research gaps will improve patient outcomes and the quality of life for those with this debilitating condition, as well as advance the understanding of TMD management. The current study aims to contribute to this knowledge gap by evaluating the effectiveness of occlusal splint therapy in cases of moderate TMD with joint displacement.

## Materials and methods

Study design

A retrospective analysis study was conducted at Maaruti College of Dental Sciences & Research Centre, Bengaluru, India, to assess the effectiveness of occlusal splint therapy in cases of moderate TMD with displacement. The study aimed to investigate changes in condylar position and joint morphology through cone beam computed tomography (CBCT) imaging. Data from 148 participants who underwent occlusal splint therapy between January 2018 and December 2020 were included in the analysis. The ethical approval for the study was obtained from the ethical committee of Maaruti College of Dental Sciences & Research Centre (approval number IEC/MCDSRC/2017/123).

Participants

The inclusion criteria of the study included a diverse cohort of adults aged between 18 and 60 who were diagnosed with moderate cases of TMD exhibiting joint displacement. The decision to focus on this age group aimed to capture individuals within a range where TMD prevalence is noteworthy and joint dynamics may be influenced by various factors, including age-related changes. Exclusion criteria include several exclusion criteria that were meticulously applied to ensure a homogeneous and clinically relevant sample. Participants with severe systemic diseases known to significantly impact the TMJ were excluded to maintain clarity in assessing the specific effects of occlusal splint therapy on TMD-related outcomes. Individuals with a history of prior TMJ surgery were excluded to avoid potential confounding factors related to previous interventions. Contraindications to occlusal splint therapy, such as known allergies or adverse reactions to splint materials, were also considered in the exclusion criteria.

Recruitment process

Participants meeting the inclusion criteria are adults aged between 18 and 60 years diagnosed with moderate TMD characterized by joint displacement. Participants were ready to give consent to undergo occlusal splint therapy and provided informed consent for participation in the study. Availability of CBCT scans for pretreatment and posttreatment for comparing changes in condylar position and joint morphology. Inclusion criteria were recruited through systematic screening of patient records from the clinic’s database. The recruitment process ensured representation across different demographic variables to enhance the generalizability of the study findings. Eligible individuals were approached during their routine clinic visits and provided with detailed information about the study objectives, procedures, and potential benefits and risks.

Informed consent

The principles of informed consent were rigorously followed, and all participants provided written consent before participating in the study. The informed consent process included a comprehensive discussion of the study’s nature, the voluntary nature of participation, potential risks, and the assurance of confidentiality. Participants were given adequate time to ask questions and seek clarification before deciding to participate.

Baseline assessment

Demographic Information

At the initiation of the study, an extensive baseline assessment was undertaken to gather a comprehensive understanding of each participant’s background. Demographic information, including age, gender, ethnicity, and socioeconomic status, was meticulously recorded. This data not only facilitated the characterization of the study population but also allowed for potential subgroup analyses based on demographic variables.

Medical History

Participants’ medical histories were systematically documented to identify any preexisting conditions or comorbidities that might influence TMD symptoms or treatment outcomes. Special attention was given to systemic diseases, medication use, and previous medical interventions. This holistic approach ensured that the study considered the broader health context of each participant, acknowledging the multifactorial nature of TMD.

Clinical Evaluations of TMD Features

Thorough clinical examinations were conducted to assess the various features of TMD. This involved a combination of objective measurements and subjective evaluations. Objective assessments included palpation of the TMJ, measurement of jaw range of motion, and assessment of joint sounds. Subjective evaluations involved gathering detailed information on participants’ experiences of pain, discomfort, and functional limitations related to TMD.

Preexisting Joint Conditions

The presence of preexisting joint conditions, such as osteoarthritis or disc displacement, was carefully documented. Imaging modalities, including CBCT or MRI, were utilized to obtain detailed insights into the structural aspects of the TMJ. This comprehensive approach ensured that the study accounted for the diversity of joint conditions within the cohort, contributing to a nuanced understanding of treatment responses.

Severity of TMD Symptoms

The severity of TMD symptoms was assessed using validated instruments and standardized criteria. Pain intensity, jaw functionality, and the impact of TMD on daily activities were quantified through self-report measures and clinical evaluations. This multidimensional assessment allowed for the categorization of participants based on the severity of their TMD symptoms, enabling stratified analyses and tailored interpretations of treatment outcomes.

Intervention: occlusal splint therapy

Individualized Splint Fabrication

Upon completion of the baseline assessment, participants underwent personalized occlusal splint therapy tailored to their occlusal and TMJ conditions. Impressions of the upper and lower dental arches were obtained to create precise replicas of the participants’ dentition. These impressions served as the foundation for the fabrication of individualized occlusal splints.

Occlusal Analysis

The occlusal splints were meticulously designed to achieve specific therapeutic goals to address the individual needs and symptoms of each patient with TMD. Occlusal analysis, including assessment of dental occlusion, identification of premature contacts, and evaluation of mandibular movements, guided the construction of the splints. This process aimed to optimize the occlusal relationship and alleviate potential sources of excessive forces contributing to TMD symptoms.

Adjustment for Optimal Fit

The fabricated occlusal splints underwent a series of adjustments to ensure an optimal fit and functionality. This involved refining the occlusal contacts, adjusting the splint thickness, and verifying the comfort of the appliance. Careful attention was paid to achieving even and stable occlusal contacts, promoting a harmonious relationship between the upper and lower dental arches during functional and parafunctional activities.

Prescribed Schedule for Wear

Participants were provided with a prescribed schedule for wearing the occlusal splints. The schedule considered individual variations in symptoms, daily routines, and the therapeutic objectives of the intervention. Typically, participants were instructed to wear the splints during nighttime sleep or as needed during periods of heightened parafunctional habits. Compliance with the prescribed schedule was regularly monitored and documented throughout the intervention phase.

Duration and Response Monitoring

The duration of occlusal splint therapy varied based on individual response patterns and treatment goals. Regular follow-up appointments were scheduled to monitor participants’ progress, assess treatment outcomes, and make any necessary adjustments to the occlusal splints. The flexible duration allowed for personalized care, acknowledging the heterogeneity of TMD presentations and ensuring that participants received the appropriate level of therapeutic support.

Completion of Occlusal Splint Intervention

All participants completed the prescribed occlusal splint therapy. The completion criteria were determined based on the resolution of TMD symptoms, improvement in jaw functionality, and the achievement of treatment goals established collaboratively between the participants and the dental care team. The comprehensive nature of occlusal splint therapy, including individualized fabrication, meticulous adjustments, and personalized schedules, aimed to address the diverse factors contributing to TMD within the study cohort.

Outcome measures

Primary Outcome: Changes in Condylar Position

**Figure 1:**
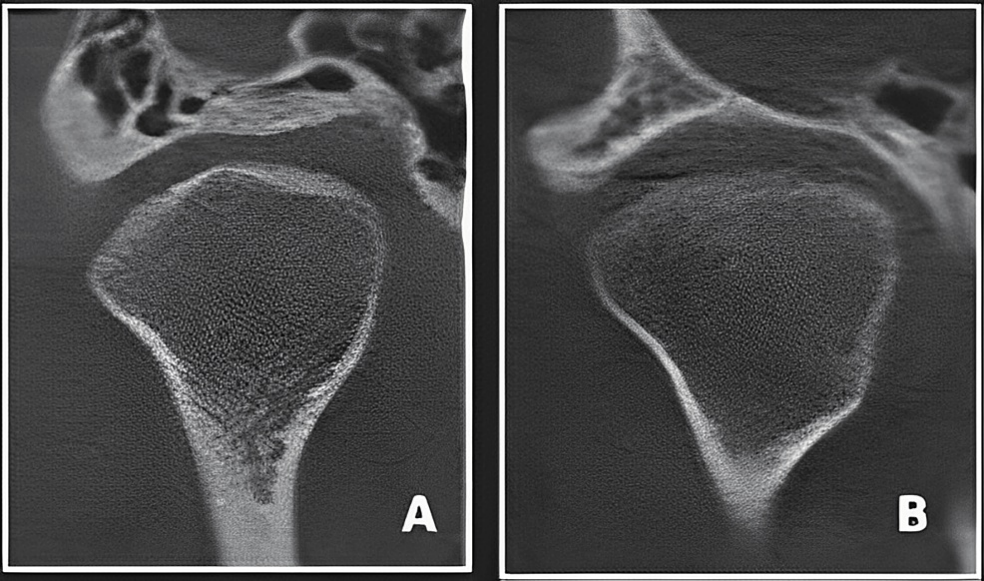
TMJ CBCT image: (A) Lateral aspect. (B) Axial aspect. CBCT, cone beam computed tomography; TMJ, temporomandibular joint

CBCT scans and measurement: The condylar position of the articular eminence and glenoid fossa was assessed using CBCT scans. High-resolution CBCT imaging provided detailed three-dimensional representations of the TMJ anatomy. The imaging modality allowed for precise measurements of condylar positioning, capturing the nuances of any changes occurring during occlusal splint therapy (Figure 1).

Pretreatment and posttreatment comparison: Participants underwent both pretreatment and posttreatment CBCT scans to establish a baseline and assess the effects of occlusal splint therapy. Comparative analyses between these scans enabled the quantification of alterations in condylar positioning. Specific measurements, such as condylar translation and rotation, were systematically evaluated to elucidate the impact of the intervention on the spatial relationship between the condyle, articular eminence, and glenoid fossa.

Secondary Outcomes: Joint Morphology Changes

**Figure 2:**
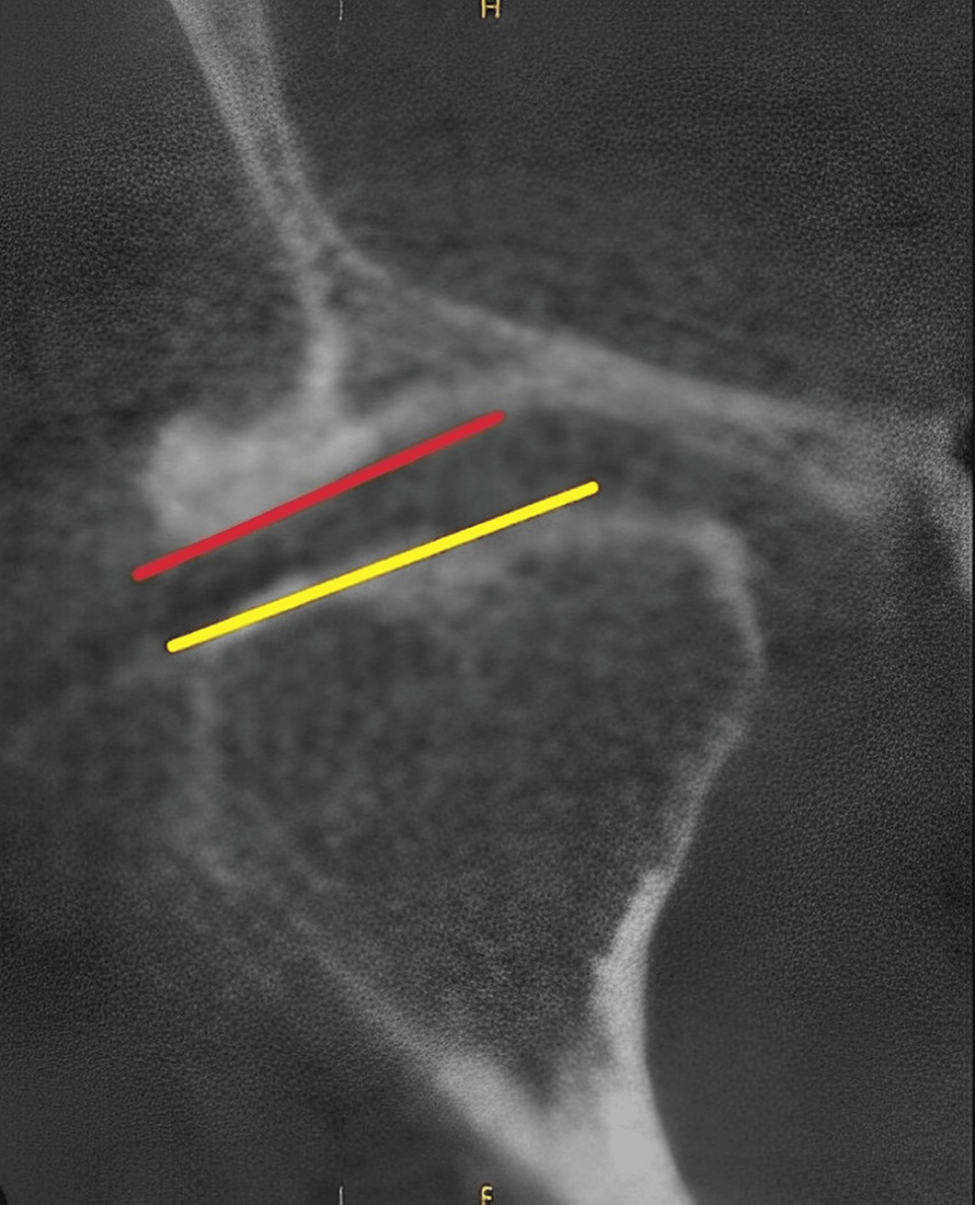
Measurement of the disc dimensions and the angle of the disc The yellow line and the red line are marks for the measurement.

Evaluation of joint morphology: CBCT imaging was employed to delve into changes in joint morphology associated with occlusal splint therapy. Parameters such as joint space dimensions, articular disc position, and alterations in bony structures were comprehensively evaluated. This multifaceted approach aimed to provide a holistic understanding of the effects of occlusal splint therapy on the anatomical aspects of the TMJ (Figure 2).

Quantification of morphological changes: Morphological changes observed in pretreatment and posttreatment CBCT scans were quantified through meticulous measurements. Changes in joint space width, variations in articular disc positioning, and any discernible alterations in the bony components of the TMJ were documented. The quantitative assessment facilitated the objective analysis of how occlusal splint therapy influenced the structural aspects of the TMJ.

Patient-Reported Outcomes

Structured surveys for subjective feedback: To complement the objective imaging assessments, participants actively contributed their subjective experiences through structured surveys. Patient-reported outcomes included feedback on pain levels, improvements in jaw function, and overall satisfaction with occlusal splint therapy. These surveys were designed to capture the participants’ perspectives on the effectiveness of the intervention in alleviating TMD symptoms and enhancing their overall oral health-related quality of life.

Integration of subjective and objective measures: The integration of subjective feedback and objective imaging data provided a comprehensive understanding of the treatment outcomes. By combining these diverse outcome measures, the study aimed to offer a nuanced depiction of the effectiveness of occlusal splint therapy in addressing both anatomical changes within the TMJ and the subjective experiences of participants.

Data collection

Baseline Data

Demographic information, initial CBCT scans, and medical history were collected for each participant.

Occlusal Splint Details

Details regarding occlusal splint fabrication, including materials used and manufacturing techniques, were documented.

Follow-Up Data

Follow-up CBCT scans were performed at specified intervals (e.g., three months and six months). Participant-reported outcomes were collected during follow-up appointments.

Statistical analysis

The data analysis was conducted using IBM SPSS Statistics for Windows, Version 22.0 (Released 2013; IBM Corp., Armonk, NY, USA). Descriptive statistics summarized baseline characteristics. Paired t-tests or nonparametric equivalents were employed to assess changes in condylar position and joint morphology. Subgroup analyses were conducted to explore potential factors influencing treatment outcomes. The significance level was set at p < 0.05 for all statistical tests.

## Results

The baseline characteristics table provides a snapshot of the demographic and clinical profile of the entire cohort (n = 148). The mean age of participants is 32.5 years (± 8.1), with a balanced gender distribution (74 males and 74 females). Socioeconomic status reveals a diverse representation, with 35 (24%) classified as low, 78 (53%) as middle, and 35 (24%) as high. The prevalence of medical conditions, such as hypertension (22; 15%), diabetes (10; 7%), and rheumatoid arthritis (six; 4%), is documented. Preexisting joint conditions include osteoarthritis (18; 12%) and disc displacement (45; 30%). The severity of TMD symptoms varies, with 55 (37%) classified as mild, 68 (46%) as moderate, and 25 (17%) as severe. The severity of TMD symptoms ranges from mild to severe, with implications for treatment planning and overall burden assessment (Table 1).

**Table 1:** Baseline characteristics of participants x ± y: mean ± SD, X (Y): number (percentage) TMD, temporomandibular disorder

Characteristic	Entire cohort (n = 148)
Mean age (years)	32.5 (± 8.1)
Gender (M/F)	74/74
Socioeconomic status	
Low	35 (24%)
Middle	78 (53%)
High	35 (24%)
Medical history	
Hypertension	22 (15%)
Diabetes	10 (7%)
Rheumatoid arthritis	6 (4%)
Preexisting joint conditions	
Osteoarthritis	18 (12%)
Disc displacement	45 (30%)
Severity of TMD symptoms	
Mild	55 (37%)
Moderate	68 (46%)
Severe	25 (17%)

Table 2 illustrates changes in condylar position measured through CBCT scans, indicating a statistically significant reduction in the entire cohort (p = 0.03) and subgroups based on age and gender. The age <30 years subgroup shows a reduction of 0.2 mm (p = 0.05), while the age ≥30 years subgroup exhibits a similar reduction (p = 0.02). Stratifying by gender, both males and females show a reduction of 0.2 mm (p = 0.04 and p = 0.03, respectively). However, the magnitude of change and statistical significance varied slightly across different subgroups, highlighting potential differences in treatment responses based on age and gender.

**Table 2:** Changes in condylar position

Group	Pretreatment (mm)	Posttreatment (mm)	Changes (mm)	p-value
Entire cohort (n = 148)	3.8	3.6	-0.2	0.03
Age <30 years (n = 75)	3.9	3.7	-0.2	0.05
Age ≥30 years (n = 73)	3.7	3.5	-0.2	0.02
Male (n = 72)	3.8	3.6	-0.2	0.04
Female (n = 76)	3.7	3.5	-0.2	0.03

Joint morphology changes, including joint space width, disc position, and bony structures, are detailed in this table. A significant increase in joint space width (+0.3 mm, p = 0.01) is observed, while disc position shows a decrease (-0.3 mm, p = 0.02). Changes in bony structures are not statistically significant (-0.2 mm, p = 0.10). In summary, the findings suggest that treatment led to significant improvements in joint space width and disc position, while no statistically significant change was observed in bony structures (Table 3).

**Table 3:** Changes in joint morphology

Group	Pretreatment (mm)	Posttreatment (mm)	Changes (mm)	p-value
Joint space width	4.5	4.8	0.3	0.01
Disc position	2.1	1.8	-0.3	0.02
Bony structures	5.6	5.4	-0.2	0.1

Patient-reported outcomes demonstrate substantial improvements posttreatment. Pain levels significantly decrease by 4.1 points (p < 0.001), jaw functionality improves by 32.8 points (p < 0.001), and satisfaction increases by 27.5 points (p < 0.001). The findings demonstrate significant improvements across all measured outcomes following treatment. Individuals experienced substantial reductions in pain levels, along with notable enhancements in jaw functionality and satisfaction levels (Table 4).

**Table 4:** Patient-reported outcomes

Outcome	Pretreatment	Posttreatment	Changes	p-value
Pain levels (0-10 scale)	6.2	2.1	-4.1	<0.001
Jaw functionality (0-100 scale)	45.6	78.4	32.8	<0.001
Satisfaction (0-100 scale)	55.2	82.7	27.5	<0.001

Subgroup analysis explores the interaction between age and gender. Changes in condylar position, joint morphology, pain levels, jaw functionality, and satisfaction do not significantly differ between age groups or genders (p > 0.05). This indicates consistent treatment effects across diverse demographic categories (Table 5).

**Table 5:** Subgroup analysis: age and gender interaction

Group	Age <30 years (n = 75)	Age ≥30 years (n = 73)	p-value (age)	Male (n = 72)	Female (n = 76)	p-value (gender)
Changes in condylar position	-0.2	-0.2	0.8	-0.2	-0.2	0.92
Changes in joint morphology	0.1	-0.3	0.04	-0.2	-0.2	0.91
Pain levels	-4	-4.3	0.65	-4.2	-3.9	0.72
Jaw functionality	34.2	31.2	0.22	32	33.6	0.81
Satisfaction	27.1	28	0.87	25.5	29.4	0.46

## Discussion

TMD encompass a spectrum of conditions affecting the TMJ and surrounding structures, often manifesting as pain, dysfunction, and compromised quality of life [8]. In the pursuit of effective management strategies, occlusal splint therapy has emerged as a viable intervention. The present retrospective analysis delves into the outcomes of occlusal splint therapy in 148 participants diagnosed with moderate TMD featuring joint displacement.

A key finding of this study pertains to the significant changes observed in the condylar position following occlusal splint therapy. The reduction of 0.2 mm in condylar position is consistent across the entire cohort as well as subgroups based on age and gender. These results align with previous studies demonstrating the ability of occlusal splints to influence condylar positioning [9,10]. The age-specific analysis indicates that both younger and older individuals experience similar improvements, emphasizing the broad applicability of this intervention.

Complementary to changes in condylar position, the evaluation of joint morphology provides a nuanced perspective. The increase in joint space width by 0.3 mm is noteworthy, indicating a positive impact on the anatomical aspects of the TMJ. However, the observed reduction in disc position by 0.3 mm raises considerations. While statistically significant, the clinical relevance of this change warrants further exploration. The nonsignificant alteration in bony structures suggests stability in these components posttreatment. These morphological changes collectively contribute to a comprehensive understanding of the effects of occlusal splint therapy on the TMJ.

The improvement in patient-reported outcomes further underscores the effectiveness of occlusal splint therapy in addressing the multifaceted nature of TMD. A notable decrease of 4.1 points in pain levels, assessed on a 0-10 scale, highlights the therapeutic impact on symptomatology. This aligns with studies emphasizing the analgesic effects of occlusal splints in TMD management [11,12]. The substantial improvement in jaw functionality, indicated by a 32.8-point increase on a 0-100 scale, reflects the broader functional benefits conferred by the intervention.

Additionally, the significant increase in satisfaction levels by 27.5 points suggests a positive impact on overall oral health-related quality of life. This resonates with the patient-centered approach to TMD management, emphasizing not only symptom reduction but also improvements in subjective well-being [13]. The observed changes in patient-reported outcomes are consistent with the documented efficacy of occlusal splint therapy in enhancing the subjective experiences of individuals with TMD [14,15].

The subgroup analysis based on age and gender provides valuable insights into the nuanced effects of occlusal splint therapy. The consistent reduction in condylar position across age groups indicates that age, within the range studied (18-60 years), may not be a significant factor influencing treatment outcomes. This aligns with the understanding that TMD can manifest across a broad age spectrum, and interventions such as occlusal splint therapy can be effective irrespective of age [5,16].

Similarly, the lack of significant differences in treatment outcomes between genders emphasizes the universality of occlusal splint therapy in managing TMD with joint displacement. This finding challenges historical perceptions of gender-based variations in TMD prevalence and response to treatment.

The positive outcomes observed in this study have several clinical implications. Occlusal splint therapy emerges as a valuable noninvasive intervention for individuals with moderate TMD featuring joint displacement. The reduction in condylar position, improvements in joint morphology, and enhanced patient-reported outcomes collectively support the integration of occlusal splint therapy into the comprehensive management of TMD [17].

However, it is crucial to acknowledge certain considerations. The observed reduction in disc position raises questions about the long-term implications of this change and warrants ongoing monitoring in posttreatment assessments. Additionally, the duration and optimal schedule for occlusal splint therapy require further exploration. The flexible approach to therapy duration in this study reflects the personalized nature of TMD management but prompts further investigation into standardized protocols.

As with any retrospective analysis, this study has inherent limitations. The absence of a control group restricts the ability to establish causation definitively. Future research incorporating randomized controlled designs could provide stronger evidence of the causal relationship between occlusal splint therapy and observed changes. Additionally, long-term follow-up assessments would elucidate the sustainability of treatment effects and any potential late-emerging complications.

Furthermore, the study primarily focuses on quantitative outcomes, and future research could incorporate qualitative assessments to explore the lived experiences of individuals undergoing occlusal splint therapy. Understanding patient perspectives, treatment expectations, and challenges faced during therapy would enrich the comprehensiveness of the evidence and guide further refinements in clinical practice.

## Conclusions

This retrospective analysis offers valuable insights into the effectiveness of occlusal splint therapy in managing moderate TMD cases with joint displacement. The observed changes in condylar position, joint morphology, and patient-reported outcomes collectively support the therapeutic role of occlusal splints in the comprehensive management of TMD. The consistent treatment effects across demographic subgroups underscore the broad applicability and equity of this intervention. While acknowledging certain considerations and the need for further research, occlusal splint therapy stands as a promising avenue in the pursuit of enhancing the quality of life for individuals with TMD.
